# Variantes genéticas recurrentes y priorización de variantes de significado clínico desconocido asociadas al síndrome de cáncer de mama y ovario hereditario en familias de la Región de Murcia

**DOI:** 10.1515/almed-2023-0032

**Published:** 2023-07-10

**Authors:** Laura Rosado-Jiménez, Younes Mestre-Terkemani, Ángeles García-Aliaga, Miguel Marín-Vera, José Antonio Macías-Cerrolaza, María Desamparados Sarabia-Meseguer, María Rosario García-Hernández, Marta Zafra-Poves, Pilar Sánchez-Henarejos, Francisco Ayala de la Peña, José Luis Alonso-Romero, José Antonio Noguera-Velasco, Francisco Ruiz-Espejo

**Affiliations:** Laboratorio de Genómica, Servicio de Análisis Clínicos, Hospital Clínico Universitario Virgen de la Arrixaca, Murcia, España; Servicio de Oncología Médica, Hospital Clínico Universitario Virgen de la Arrixaca, Murcia, España; Servicio de Oncología Médica, Hospital General Universitario Morales Meseguer, Murcia, España

**Keywords:** efecto fundador, espectro mutacional, priorización, síndrome de cáncer de mama y ovario hereditario, variantes de significado clínico desconocido, variantes patogénicas recurrentes

## Abstract

**Objetivos:**

El síndrome de cáncer de mama y ovario hereditario (SCMOH) presenta un patrón de herencia autosómica dominante en genes de susceptibilidad al cáncer y su riesgo está principalmente vinculado a mutaciones germinales en *BRCA1* y *BRCA2*. Sin embargo, la implementación de paneles genéticos mediante secuenciación masiva en la práctica asistencial, ha ampliado el espectro mutacional de este síndrome hereditario y el número de variantes de significado clínico desconocido (VUS) detectadas en los estudios genéticos.

**Métodos:**

El estudio de prevalencia del SCMOH realizado en 2928 familias de la Región de Murcia ha permitido identificar las variantes patogénicas recurrentes y mutaciones fundadoras, principalmente asociadas a genes *BRCA1* y *BRCA2*. En el estudio de VUS destaca la aplicación de un algoritmo de priorización diseñado por nuestro grupo de trabajo.

**Resultados:**

Las variantes c.68_69del, c.212+1G>A, y c.5123C>A fueron detectadas en un 30 % de los portadores de *BRCA1* mientras que la deleción del exón 2 junto con c.3264dupT, c.3455T>G y c.9117G>A se han encontrado en un 30 % de los portadores de *BRCA2*. Un total de 16 VUS (15 %) fueron priorizadas.

**Conclusiones:**

La correlación genotipo-fenotipo resultó compatible con lo reportado previamente en la literatura científica. Además, se ha constatado el efecto fundador de c.1918C>T (*BRCA1*) y c.8251_8254del (*ATM*) en población murciana y la deleción del exon2 (*BRCA2*) como mutación fundadora española. La implementación del algoritmo ha permitido priorizar aquellas VUS susceptibles de patogenicidad en las que sería recomendable realizar estudios complementarios para así, poder determinar su efecto clínico y su posible implicación en el SCMOH.

## Introducción

Los datos actualizados por la Agencia Internacional de Investigación del Cáncer (IARC, *International Agency for Research on Cancer*) reflejan un cambio de tendencia en la epidemiología del cáncer al situar en primera posición al cáncer de mama como el tumor más diagnosticado (11,7 %) a nivel mundial durante el año 2020 [1], representando el 24,5 % de la incidencia global en mujeres. Este tumor es responsable de la primera causa de muerte por cáncer en mujeres a nivel mundial (15,5 %) y la segunda en nuestro país (14,6 %) [2].

Por otro lado, el cáncer de ovario es el tercer tumor ginecológico más frecuente a nivel mundial, representando un 3,4 % de la incidencia global en mujeres y un 4,7 % de las muertes por cáncer en población femenina [2].

Aproximadamente, el 7 % de los tumores mamarios y el 11–15 % de los tumores epiteliales de ovario se consideran de tipo hereditario asociados a mutaciones germinales en genes de susceptibilidad al cáncer, principalmente *BRCA1* y *BRCA2* [3]. Los avances científicos relacionados con el estudio genético del SCMOH han permitido conocer nuevos genes de penetrancia variable y accionabilidad clínica demostrada para este tipo de tumores, además de otros genes cuya validez clínica todavía se encuentra en fase de investigación [4, 5].

El diseño de tecnologías de secuenciación de nueva generación (NGS, *Next Generation Sequencing*) y la reciente incorporación de paneles genéticos a la rutina diagnóstica han supuesto una revolución de gran utilidad en el diagnóstico molecular del SCMOH. No obstante, la interpretación de los resultados obtenidos puede suponer un importante desafío para los profesionales del laboratorio debido al incremento significativo de nuevos genes analizados y del número de VUS detectadas que se traducen en resultados no informativos al no poder concluir la relevancia clínica de estas variantes causando complicaciones adicionales en el asesoramiento genético [6]. Por esta razón, se debe limitar la inclusión de genes a aquellos con accionabilidad clínica demostrada, además de realizar una revisión periódica de las VUS detectadas y un seguimiento de los portadores a la espera de nuevos hallazgos que aporten evidencia científica para su futura clasificación clínica.

Del mismo modo, destaca la importancia de establecer criterios de priorización basados en la accionabilidad clínica de los genes, así como en la predicción estimada *in silico* y la información consultada en las fuentes bibliográficas disponibles. La priorización de variantes permite identificar aquellas VUS susceptibles de patogenicidad en las que realizar estudios adicionales con el fin de conocer su impacto biológico y su posible implicación en el SCMOH de forma prioritaria con respecto al resto de VUS [7].

Por otro lado, la identificación de variantes patogénicas recurrentes y fundadoras relacionadas con el cáncer de mama y ovario hereditario permite conocer el perfil mutacional asociado a una población concreta para así, diseñar el tipo de estudio genético más eficiente en cada grupo poblacional y ofrecer un asesoramiento genético especializado.

De esta forma, Rebbeck y colaboradores reportaron una descripción mundial de la prevalencia y espectro mutacional de los principales genes de predisposición genética *BRCA1/2* agrupada por origen geográfico y por grupo racial/etnia (véase Material Suplementario, Tablas 1 y 2). A pesar de que existe cierta diversidad genética en función de la población de estudio, los datos revelan que las variantes genéticas más comunes en todas las regiones del mundo fueron las conocidas como mutaciones fundadoras en judíos ashkenazi: c.68_69del (BIC: 185delAG), c.5266dup (BIC: 5382insC) en *BRCA1* y c.5946del (BIC: 6174delT) en *BRCA2* [8].

En España, el espectro mutacional de *BRCA1/2* muestra una variación considerable en las diferentes subpoblaciones españolas. No obstante, destaca un espectro de variantes patogénicas recurrentes en familias españolas con SCMOH. En relación a *BRCA1*, c.211A>G representa la variante genética más prevalente en población española (especialmente en el noroeste de España) [9] seguida de c.68_69del y c.5123C>A, ambas relacionadas con la presencia histórica de los judíos (sefardíes) en la península Ibérica por lo que se encuentran ampliamente distribuidas por todo el territorio nacional [10, 11]. A continuación, se posicionan c.3770_3771del y c.3331_3334del entre las variantes patogénicas reportadas de forma recurrente en población española. Respecto a la variante c.3331_3334del, el análisis de haplotipos realizado en portadores hispanos apunta que esta mutación ancestral fue originada en la península ibérica y posteriormente, propagada durante la colonización hacia Latinoamérica [12]. Con respecto a *BRCA2*, c.2808_2811del y c.6275_6276del se encuentran ampliamente distribuidas por diferentes subpoblaciones españolas. La diversidad de haplotipos relacionados con la variante c.2808_2811del, sugiere que posiblemente presente múltiples orígenes independientes. Sin embargo, el análisis de haplotipo realizado sobre la variante c.6275_6276del, reportada en diversas poblaciones del mundo, estima su origen en Europa del Norte [13]. Por el contrario, las variantes genéticas c.3264dup, c.9026_9030del y c.9018C>A destacan de forma recurrente en la región mediterránea [14].

## Materiales y métodos

### Selección de familias de alto riesgo

Entre abril de 2007 y abril de 2022, se seleccionaron 2928 familias procedentes de la Región de Murcia, que cumplían criterios de alto riesgo establecidos por la Sociedad Española de Oncología Médica (SEOM) y el Comité de Asesoramiento Genético de la Región de Murcia para la indicación del estudio genético del SCMOH [3, 15] (Material Suplementario, Tabla 3).

### Análisis genético del SCMOH en la Región de Murcia

**Figura 1:**
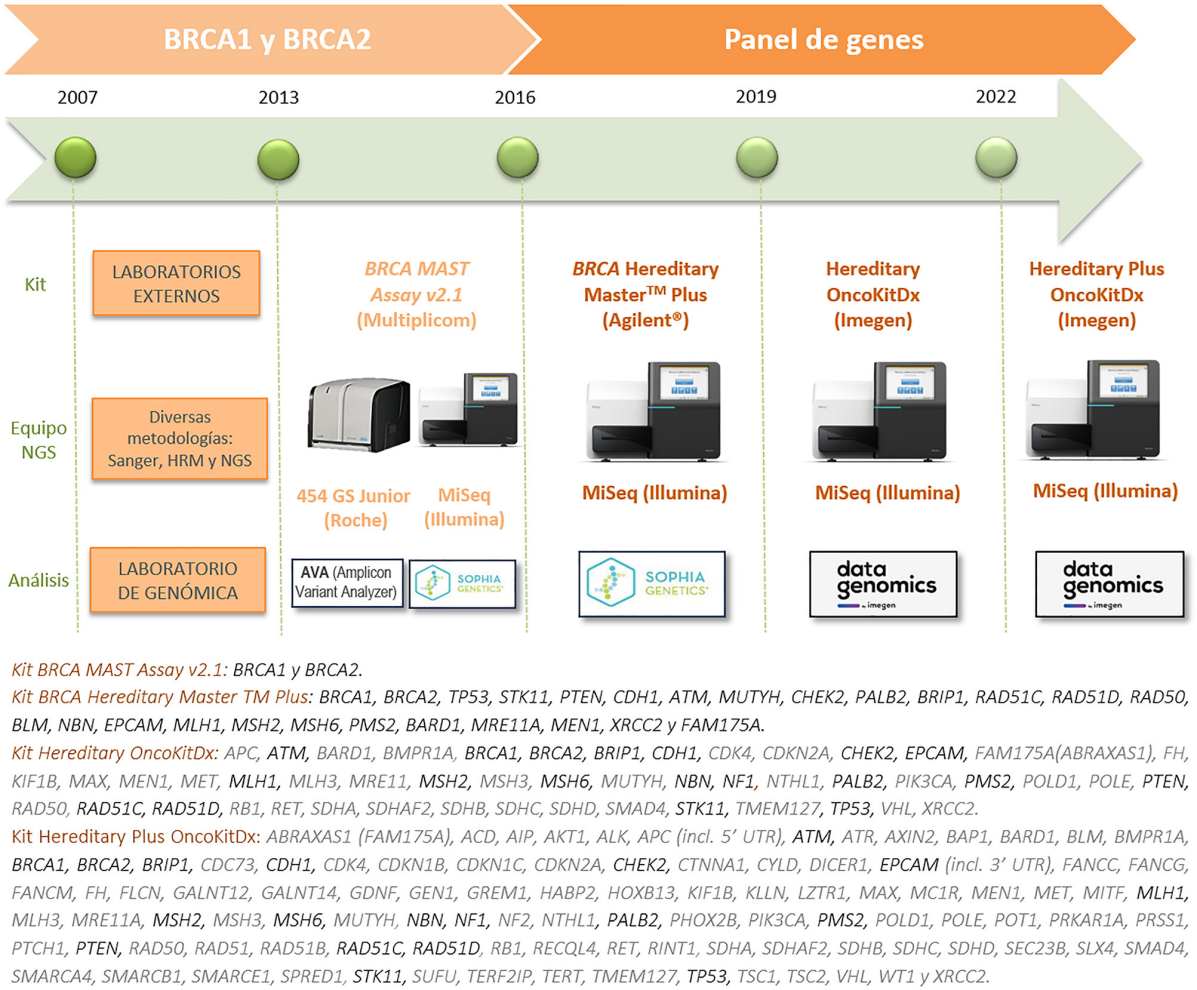
Evolución cronológica de la metodología empleada en el diagnóstico molecular del SCMOH. En negrita se destacan los genes seleccionados para el estudio bioinformático asistencial (Kit Hereditary OncokitDx y Kit Hereditary Plus OncokitDx) de acuerdo a las recomendaciones de la NCCN v.3.2019.

El estudio genético del SCMOH se realizó según la metodología ilustrada en la Figura 1 que muestra la evolución cronológica del diagnóstico molecular de este síndrome hereditario comprendida desde el análisis de *BRCA1* y *BRCA2* hasta la secuenciación masiva de un panel que incluye los genes con accionabilidad clínica demostrada para el cáncer de mama y ovario (*BRCA1, BRCA2, TP53, PTEN, CDH1, STK11, PALB2, ATM, CHEK2, NBN, NF1, BRIP1, RAD51C, RAD51D, MSH2, MLH1, MSH6, PMS2, EPCAM*) recomendados por la *National Comprehensive Cancer Network* (NCCN v.3.2019) [16].

Tras la realización del análisis genético, todas las variantes genéticas detectadas se clasificaron siguiendo los criterios de consenso recomendados por el Colegio Americano de Genética Médica y Genómica (ACMG, *American College of Medical Genetics and Genomics*) con ayuda de bases de datos y fuentes bibliográficas disponibles [17].

### Espectro mutacional del SCMOH en la Región de Murcia

El estudio diseñado para conocer el perfil mutacional del SCMOH permitió identificar las variantes clínicamente relevantes detectadas de forma recurrente en el estudio genético de este síndrome hereditario en 2928 familias de la Región de Murcia.

Se seleccionaron aquellas variantes clasificadas como patogénicas según ACMG que presentaron una elevada prevalencia, estableciendo como valor mínimo para la representación de nuestra población de estudio, un total de 10 familias por variante estudiada correspondientes a un porcentaje superior al 5 % de la población portadora *BRCA1/BRCA2*.

### Estudio de correlación genotipo-fenotipo

En las familias portadoras de una variante patogénica recurrente, se realizó un estudio de correlación genotipo-fenotipo en función de la edad del diagnóstico del tumor, procedencia geográfica, tipo de cáncer diagnosticado y sus características histopatológicas e inmunohistoquímicas; así como una comparativa de prevalencia y expresión fenotípica con otros estudios españoles e internacionales relacionados con el SCMOH.

### Análisis estadístico

El análisis de estadística descriptiva e inferencial de las variables de estudio se realizó utilizando el software SPSS v.27.

– Para el análisis descriptivo de las variables cuantitativas se utilizaron medidas de tendencia central y de dispersión mientras que las variables cualitativas se representaron a través de tablas de frecuencia absoluta y relativa.
– El contraste de hipótesis para comparar variables cualitativas se realizó mediante el test de Chi cuadrado de Pearson. Las diferencias fueron consideradas significativas cuando el valor ¨p¨ asociado a la prueba estadística de contraste fue menor a 0,05.

### Variantes patogénicas con efecto fundador en la Región de Murcia

El análisis de haplotipos de las variantes genéticas con posible efecto fundador en la Región de Murcia se realizó por electroforesis capilar mediante la técnica de microsatélites empleando el Kit Type-it Microsatellite PCR (Qiagen) de acuerdo a las condiciones especificadas en **el** Material Suplementario, Tabla 4 y Figura 1.

**Figura 2:**
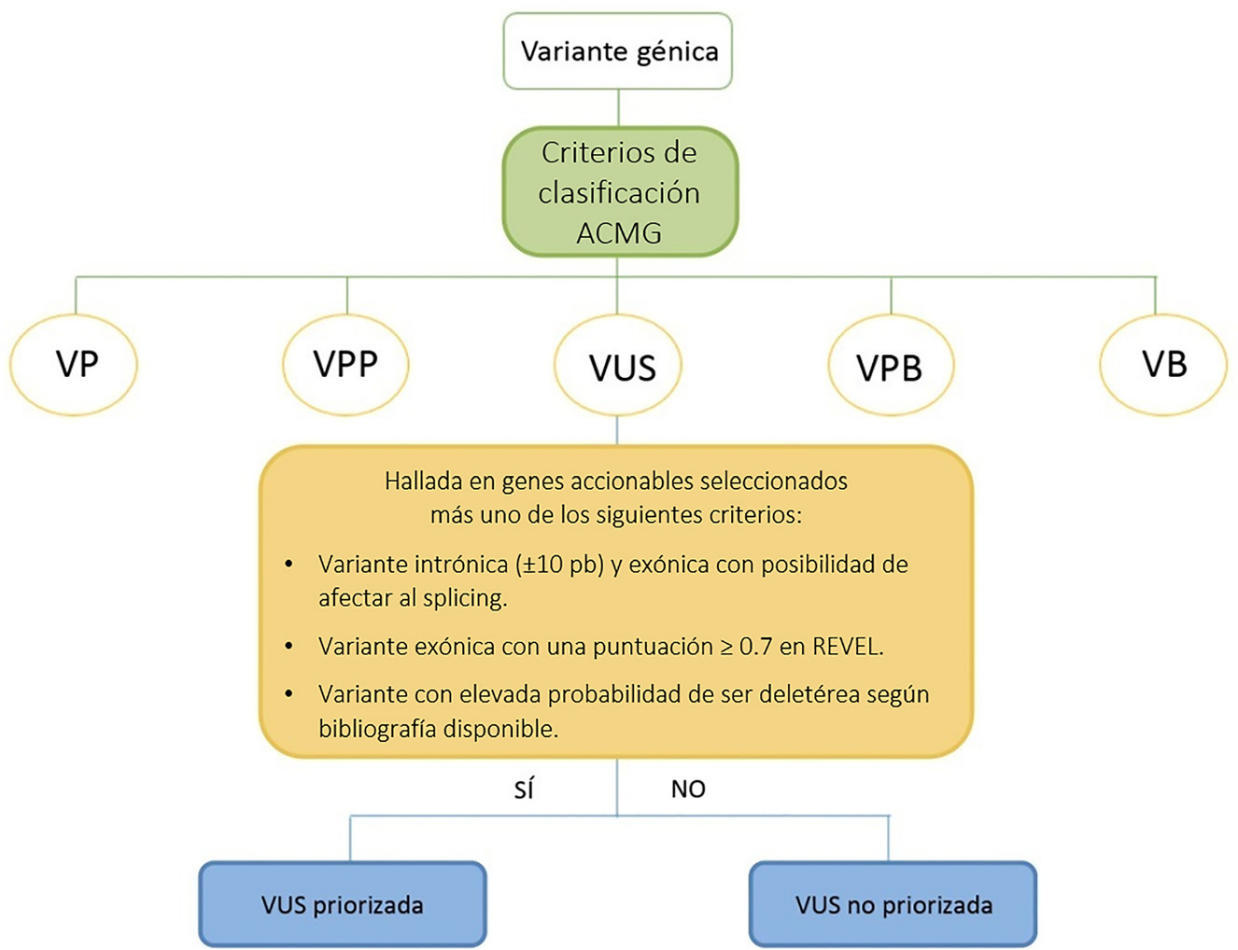
Algoritmo empleado para la priorización de VUS. VP, variante patogénica; VPP, variante probablemente patogénica; VUS, variante de significado clínico desconocido; VPB, variante probablemente benigna; VB, variante benigna; pb, pares de bases.

**Figura 3:**
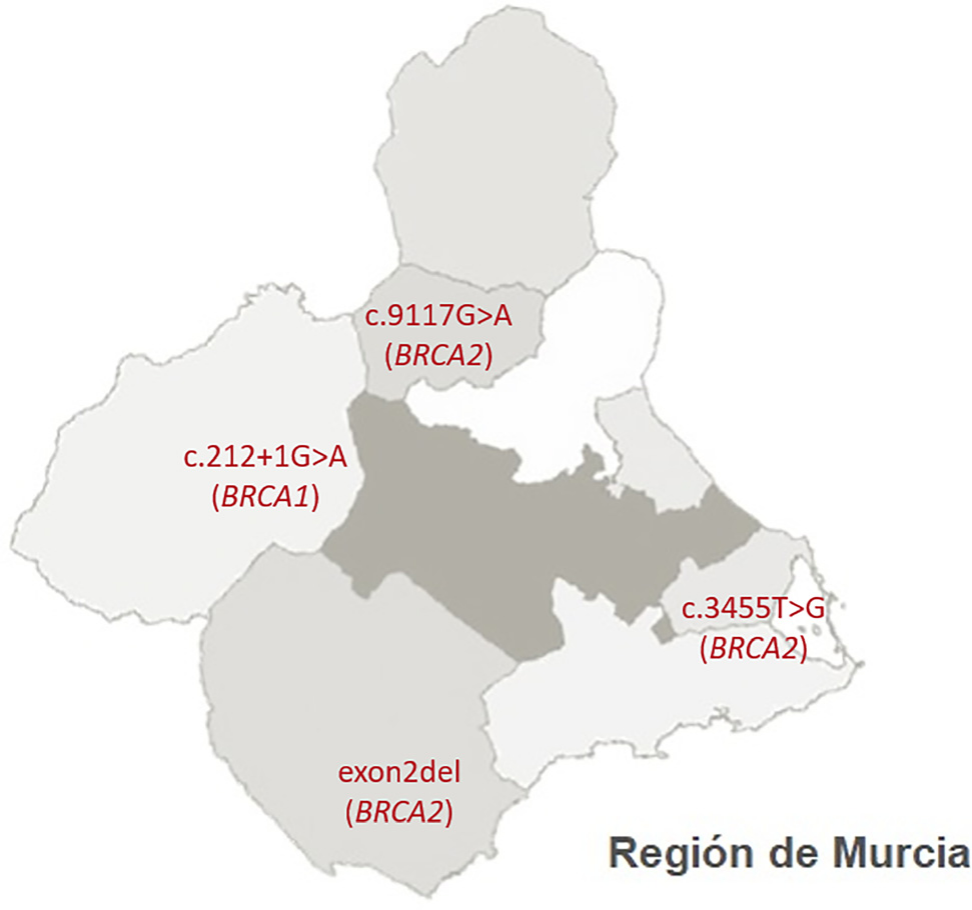
Distribución geográfica de las variantes patogénicas detectadas de forma recurrente en familias de la Región de Murcia.

Los microsatélites seleccionados para el estudio de haplotipos de las variantes c.1918C>T en *BRCA1*, deleción del exón 2 en *BRCA2* y c.8251_8254del en ATM, además de la localización de estos marcadores en los cromosomas, pueden consultarse en el Material Suplementario, Tablas 5–7 y Figuras 2–4.

De forma paralela, la técnica diseñada para cada una de las variantes fue realizada en 20 muestras control procedentes de una genoteca disponible en el Laboratorio de Genómica del Hospital Clínico Universitario Virgen de la Arrixaca (pacientes seleccionados de diferentes áreas de la Región de Murcia, sin aparente relación de parentesco, que no cumplían los criterios establecidos por la SEOM) con el fin de demostrar que el haplotipo común no se encontraba en población control, así como para estimar la antigüedad de dichas variantes.

Para el cálculo de generaciones de mutaciones fundadoras, se utilizó la ecuación desarrollada por Machado y colaboradores [18]: *G*=log *δ*/log (1 − *θ*). El símbolo *δ* hace referencia a la medida del desequilibrio de ligamiento entre la variante y cada uno de los marcadores más cercanos y se calcula a partir de la prevalencia del alelo entre los afectados (Pd) y la prevalencia del alelo entre los alelos control (Pn) mediante la fórmula *δ*=(Pd − Pn)/(1 − Pn). Por otro lado, *θ* representa la fracción de recombinación entre el marcador y el gen, calculada a partir de las distancias entre los marcadores y el gen obtenidas a partir de la base de datos *Ensembl* [19].

### Estudio de variantes de significado clínico desconocido

Las VUS detectadas en el estudio genético del SCMOH fueron reevaluadas consultando diversas bases de datos y herramientas bioinformáticas, además de investigar si existían nuevas evidencias científicas en la bibliografía que permitiesen reclasificar dichas variantes.

Posteriormente, se realizó un tratamiento de las VUS reclasificadas como tal, mediante la aplicación de un algoritmo de priorización diseñado por nuestro grupo de trabajo (Figura 2).

Los criterios de priorización establecidos han permitido seleccionar aquellas VUS halladas en genes clínicamente accionables recomendados por la NCCN [16] que cumplan al menos uno de los siguientes criterios:

– Variantes intrónicas situadas en el borde exón-intrón (±10 pb) o exónicas con predicción de *splicing* aberrante en las plataformas *in silico* (*SpliceSiteFinder, MaxEntScan, NNSplice y GeneSplicer*) incluidas en *Varsome* y *Alamut* [20].
– Variantes exónicas con una puntuación ≥0,7 en REVEL *score* (*Rare Exome Variant Ensemble Learner*). Este método permite predecir la patogenicidad de variantes de tipo *missense* en base a una combinación de herramientas bioinformáticas: *MutPred, FATHMM, VEST, PolyPhen, SIFT, PROVEAN, MutationAssesor, Mutation Taster, LRT, GERP, SiPhy, phyloP y phastCons.*
La puntuación seleccionada como umbral para discriminar la predicción entre benignidad y patogenicidad fue de 0,7 mostrando una sensibilidad de 05,786 y una especificidad de 0,9556 [21].
– Aquellas variantes genéticas con elevada probabilidad de ser deletéreas según las fuentes bibliográficas disponibles en *PubMed* y *Varsome* [22, 23].

De esta forma, la priorización de VUS permite optimizar la realización de estudios complementarios (análisis de cosegregación familiar, estudios de casos-controles o ensayos funcionales) en aquellas con mayor potencial deletéreo con el fin de determinar su efecto clínico y su posible implicación en el SCMOH.

## Resultados y discusión

### Espectro mutacional del SCMOH en la Región de Murcia

El conocimiento del espectro mutacional de predisposición hereditaria al cáncer de mama y ovario ha evidenciado la variabilidad genética existente en la Región de Murcia, lo que justifica el empleo de un panel genético que permita el análisis de los genes clínicamente accionables relacionados con este síndrome hereditario.

El estudio de prevalencia realizado en 2928 familias asociadas al SCMOH ha permitido identificar las variantes patogénicas recurrentes y mutaciones fundadoras en la Región de Murcia (Tabla 1). Los resultados de nuestro estudio revelan que, la tasa mutacional más significativa de variantes clínicamente relevantes, se corresponde con los principales genes de susceptibilidad genética *BRCA1/2*, seguida de un porcentaje relevante en genes de alta y moderada penetrancia (*ATM, CHEK2, PALB2, BRIP1 y TP53*). Por tanto, los datos de nuestro estudio resultan concordantes con lo reportado en la literatura científica [24].

**Tabla 1:** Variantes recurrentes y fundadoras *BRCA1/2* en la Región de Murcia.

Gen	Exón/Intrón	HGVS	refSNP	Tipo de variante	ClinVar	Nº familias	Fenotipo predominante
*BRCA1*	2	c.68_69del	rs80357914	*Frameshift*	VP	23	CM: DI TN
	IN4-5	c.212+1G>A	rs80358042	Intrónica	VP	16	CM: DI TN
	10	c.1918C>T	rs886039981	*Nonsense*	VP	9	CM: DI TN
	17	c.5123C>A	rs28897696	*Missense*	VP	12	CM: DI TN
*BRCA2*	2	exon2del	–	LRG	VP	12	CM: DI LA
	11	c.3264dup	rs80359380	*Frameshift*	VP	10	CM: DI LA
	11	c.3455T>G	rs80358593	*Nonsense*	VP	12	CM: DI LA
	23	c.9117G>A	rs28897756	*Synonymous*	VP	16	CM: DI LA
*ATM*	56	c.8251_8254del	rs786202120	*Frameshift*	VP	8	CM: DI LA

CM, cáncer de mama; DI, ductal invasivo; HGVS, *Human Genome Variation Society*; LA/B, luminal A/B; LGR, *Large Genomic Rearrengments*; TN, triple negativo; VP, variante patogénica.

Los resultados obtenidos indican que los cambios c.68_69del, c.212+1G>A y c.5123C>A se han detectado en un 30 % de la población portadora de variantes en *BRCA1*, mientras que la deleción del exón 2 junto con los cambios c.3264dup, c.3455T>G y c.9117G>A se han encontrado en el 30 % de la población portadora de variantes en *BRCA2* [25].

### Fenotipo de las familias portadoras

La correlación genotipo-fenotipo ha mostrado diferencias estadísticamente significativas entre las variantes patogénicas *BRCA1* y *BRCA2* detectadas de forma recurrente en familias portadoras de la Región de Murcia. De esta manera, los portadores afectados de cáncer de mama asociado a variantes patogénicas en *BRCA1* han presentado un perfil histológico dominante de tipo ductal invasivo y subtipo molecular triple negativo. En cambio, el fenotipo asociado a los portadores de variantes patogénicas *BRCA2* diagnosticados de cáncer de mama, ha resultado más heterogéneo. Al igual que la mayoría de los tumores *BRCA1* han sido de tipo ductal infiltrante, sin embargo, predomina el fenotipo luminal A con positividad de receptores hormonales y negatividad para HER2. Por tanto, las características histopatológicas e inmunofenotípicas asociadas a tumores mamarios en *BRCA1/2* resultan compatibles con lo reportado previamente en la literatura científica [26]. En relación a la edad de diagnóstico del tumor, se ha observado que la mediana en portadores *BRCA1/BRCA2* afectados de cáncer de mama fue superior a los 40 años.

Asimismo, destaca un número superior de casos de cáncer de mama bilateral asociado a las variantes c.68_69del y c.5123C>A en *BRCA1* mientras que las variantes *BRCA2* presentan una mayor prevalencia de cáncer de mama en el varón. La concordancia observada en el perfil histopatológico e inmunohistoquímico de los carcinomas mamarios bilaterales de tipo sincrónico y metacrónico fue del 65 %, inferior a lo descrito en estudios previos [27]. Este dato evidencia que la expresión fenotípica del primer tumor fue relativamente predictiva del estatus de expresión del segundo tumor. Sin embargo, no siempre resulta así, lo que refuerza la necesidad de determinar el perfil histopatológico e inmunohistoquímico de cada tumor para establecer de forma adecuada el pronóstico y la decisión terapéutica.

Previsiblemente, los carcinomas mamarios han presentado diferencias más significativas en sus características fenotípicas respecto al cáncer de ovario, mostrando éste un perfil fenotípico predominante. Prácticamente la totalidad de los casos de carcinoma ovárico observados en portadoras *BRCA* han resultado de tipo seroso de alto grado, coincidiendo con lo descrito en la bibliografía [26]. Asimismo, la edad mediana de diagnóstico del cáncer de ovario fue superior a los 50 años.

### Procedencia geográfica de las familias portadoras

En el estudio realizado sobre el origen geográfico de las familias estudiadas, se ha observado como las portadoras de las variantes c.68_69del (*BRCA1*), c.5123C>A (*BRCA1*) y c.3264dup (*BRCA2*) presentan una procedencia heterogénea, encontrándose ampliamente distribuidas por la Región de Murcia.

Sin embargo, las familias portadoras del resto de variantes patogénicas recurrentes, limitan su procedencia a áreas geográficas concretas. De este modo, la deleción del exón 2 (*BRCA2*) se delimita al Valle del Guadalentín, c.212+1G>A (*BRCA1*) a la Comarca del Noroeste, c.9117G>A (*BRCA2*) a la Vega Alta del Segura y c.3455T>G (*BRCA2*) a la zona sureste de la Región de Murcia (Figura 3).

Resulta interesante destacar que las variantes genéticas caracterizadas por una distribución heterogénea en la Región de Murcia coinciden con aquellas ampliamente descritas en el territorio nacional mientras que la deleción del exón 2 (*BRCA2*) y los cambios c.212+1G>A (*BRCA1*), c.3455T>G (*BRCA2*) y c.9117G>A (*BRCA2*) son variantes distinguidas de forma predominante en la citada región.

### Comparativa de prevalencia mutacional

El análisis comparativo realizado con población europea y española, permite afirmar que los resultados obtenidos de prevalencia mutacional asociada a *BRCA1* muestran una mayor concordancia que los referentes a *BRCA2*. Resulta interesante destacar cierta afinidad genética observada especialmente con las poblaciones de la región mediterránea debido a la proximidad geográfica existente con nuestra población de estudio.

Respecto a los grandes reordenamientos, los estudios realizados a nivel mundial reportaron una mayor frecuencia en *BRCA1* respecto a *BRCA2*, coincidiendo con lo descrito en población española. Los reordenamientos genómicos en *BRCA1* representan el 2,1 % de las familias españolas con cáncer de mama y ovario hereditario, mientras que en *BRCA2* explican el 1,5 % [28, 29]. Curiosamente, los resultados de nuestro estudio contrastan con lo descrito en la bibliografía, encontrando una mayor prevalencia de familias portadoras de reordenamientos genómicos en *BRCA2* (4,1 % *BRCA1* vs 7,8 % *BRCA2*), destacando la deleción del exón 2 como el reordenamiento genómico más recurrente en la población estudiada.

### Variantes patogénicas con efecto fundador en la Región de Murcia

El análisis de microsatélites ha confirmado la presencia de un haplotipo común de dos mutaciones fundadoras en la Región de Murcia: la variante c.1918C>T (*BRCA1*) detectada de forma exclusiva en nuestra población de estudio [30] y la deleción c.8251_8254del (*ATM*) [31] (véase Material Suplementario, Tabla 8 y Figura 5). Además, se ha demostrado que ambas variantes son de reciente aparición al estimar su origen hace aproximadamente 18 y 7 generaciones, respectivamente [32].

Del mismo modo, la deleción del exón 2 (*BRCA2*) se ha constatado como variante fundadora en población española al demostrar la presencia de un ancestro común con las familias estudiadas en la publicación de Ruiz de Garibay y colaboradores [33] (véase Material Suplementario, Tabla 9). La estimación de la antigüedad de dicha variante fue de 22 generaciones, lo que supone que sea una mutación fundadora de reciente aparición [34]. Este efecto fundador acentuado en población murciana explicaría una fracción significativa de los reordenamientos genómicos detectados en *BRCA2*.

### Estudio de variantes de significado clínico desconocido

En los genes de predisposición al cáncer hereditario, la interpretación clínica de variantes genéticas resulta esencial en el proceso de asesoramiento genético de las familias portadoras. El notable incremento de VUS detectadas alerta sobre la necesidad de implementar un sistema de revisión eficiente que incluya estrategias de priorización para optimizar la realización de estudios complementarios en aquellas VUS priorizadas [35].

En nuestra población de estudio, se registraron un total de 115 VUS en genes clínicamente accionables, siendo la mayoría de tipo *missense*. Un total de 7 fueron reclasificadas como benignas o probablemente benignas y 1 como probablemente patogénica, mientras que el 93 % del total de variantes se mantuvieron como VUS a pesar de los esfuerzos empleados en el proceso de reclasificación (Tabla 2). Estos resultados coinciden con lo reportado en otros estudios de reclasificación de variantes, destacando un número superior de variantes reclasificadas como benignas [36].

**Tabla 2:** Reclasificación de VUS.

Gen	Exón/Intrón	HGVS	Tipo de variante	ClinVar	HGMD	Varsome	Criterios ACMG	Reclasificación
*BRCA1*	10	c.2634A>G	*Synonymous*	VPB	ND	VB	BP4+BP6+BP7	VPB
		c.2770A>G	*Missense*	VPB	ND	VPB	PM2, BP3+BP4	VPB
		c.3759T>A	*Synonymous*	VPB	ND	VPB	BP4+BP6+BP7	VPB
	23	c.5507_5508del	*Frameshift*	ND	DM	VPP	PVS1, PM2	VPP
*BRCA2*	IN 5	c.475+14C>T	Intrónica	VPB	ND	VPB	BP4+BP6	VPB
	IN 13	c.7008-14del	Intrónica	VPB	ND	VPB	BP6	VPB
*ATM*	IN 8	c.1066-6T>G	Intrónica	VB	VUS	VPB	BS1+BS2, BP6, PP5	VB
	IN 45	c.6572+11C>T	Intrónica	VPB	ND	VPB	BP4+BP6	VPB

DM, mutación causante de enfermedad; HGMD, *Human Gene Mutation Database*; HGVS**,** *Human Genome Variation Society*; ND, no descrita; VPP, variante probablemente patogénica; VUS, variante de significado clínico desconocido; VPB, variante probablemente benigna; VB, variante benigna.

Posteriormente, la implementación del algoritmo diseñado por nuestro grupo de trabajo permitió priorizar 16 variantes, lo que supuso un 15 % del total de VUS detectadas (Tabla 3).

**Tabla 3:** VUS priorizadas junto al fenotipo asociado a portadores y criterios de priorización.

Gen	HGVS	refSNP	Tipo de variante	Nº casos	Caso índice	Criterio de priorización	
*ATM*	c.967A>G	rs587781511	*Missense*	1	CM (47)	Bibliografía	Li A y col [37]. George Priya Doss C y col [38]. Carranza y col [39]. Fiévet A y col [40].
	c.3402+3A>C	rs786203688	Intrónica	1	CM (34)	*Splicing*	Varsome (0,9998)
	c.4388T>G	rs138327406	*Missense*	2	CO (34)	REVEL	Patogénica (0,758)
					CM (49)		
	c.6067G>A	rs11212587	*Missense*	1	CMb (39)	Bibliografía	Mangone FR y col [41]. Podralska M y col [42]. Thorstenson YR y col [43].
*BRCA2*	c.3032C>G	rs80358548	*Missense*	1	CMb (45,47)	REVEL	Patogénica (0.72)
	c.4594G>T	rs80358693	*Missense*	1	CM (31)	Bibliografía	Ochiai K y col [44].
	c.7559G>T	rs80358982	*Missense*	1	CO (25)	REVEL	Patogénica (0,7419)
	c.9275A>G	rs80359195	*Missense*	3	CM (61)	REVEL	Patogénica (0,808)
					CM (36)		
					CM (49)		
*CHEK2*	c.320-5T>A	rs121908700	Intrónica	2	CM (27)	*Splicing*	Varsome (0,8037)
					CM (37)		
	c.442A>G	rs876660482	*Missense*	1	CM (47)	Bibliografía	Delimitsou A y col [45]. Apostolou y col [46].
						REVEL	Patogénica (0,85)
	c.1008G>A	rs201544715	*Synonymous*	1	CM (38)	*Splicing*	Varsome (0,9999)
	c.1427C>T	rs142763740	*Missense*	1	CM (21)	Bibliografía	Yurgelun MB y col [47]. Eliade M y col [48]. Desrichard y col [49] Roeb W y col. [50]
*MSH2*	c.470G>C	rs765489269	*Missense*	1	CM (39)	REVEL	Patogénica (0,763)
	c.1045C>G	rs267607939	*Missense*	1	CM (42)	REVEL	Patogénica (0,9929)
*MSH6*	c.3883C>T	ND	*Missense*	1	CO (39) CM (60)	REVEL	Patogénica (0,7059)
*NBN* ^a^	c.2071-4A>G	rs746994234	Intrónica	1	CM (47)	*Splicing*	Varsome (0,9987)

^a^Una limitación destacable en el estudio de priorización de VUS fue la imposibilidad de obtener la muestra biológica necesaria para realizar el estudio *in vitro* de la variante priorizada c.2071-4A>G en *NBN* susceptible de alterar el patrón de *splicing*, tal y como se predice en los programas computacionales. CM, cáncer de mama; CMb, CM bilateral; CO, cáncer de ovario; HGVS, *Human Genome Variation Society*; ND, no descrita; REVEL, *Rare Exome Variant Ensemble Learner*.

Por último, resulta interesante destacar las evidencias científicas que apoyan la posible patogenicidad de las variantes priorizadas de acuerdo a la bibliografía disponible:

– La variante c.967A>G produce un cambio de isoleucina por valina en el residuo 323 de la proteína ATM. Aunque las herramientas bioinformáticas utilizadas proporcionan predicciones discordantes, se debería considerar su posible patogenicidad ya que se ha descrito en pacientes con ataxia telangiectasia y se ha clasificado como deletérea al afectar a la funcionalidad de la proteína [37], [38], [39], [40].
– La variante c.6067G>A produce un cambio de glicina por arginina en el residuo 2023 de la proteína ATM. La mayoría de las herramientas bioinformáticas utilizadas apoyan su patogenicidad. Esta variante fue descrita en pacientes con cáncer de mama en estudios previos, prediciéndose como potencialmente patogénica pero no se ha confirmado con estudios funcionales o de casos-controles [41], [42], [43]. Por lo tanto, se requieren estudios adicionales que aporten conclusiones sobre su efecto deletéreo y su posible asociación con un mayor riesgo de cáncer.
– La variante c.4594G>T reemplaza la valina con fenilalanina en el codón 1532 de la proteína BRCA2. La mayoría de las herramientas bioinformáticas apoyan la patogenicidad de esta variante descrita en pacientes con cáncer de mama. Los estudios experimentales han demostrado que la valina 1532 juega un papel importante en la interacción entre BRCA2 y RAD51 y, por lo tanto, la variante V1532F debilita la interacción de BRC4 con RAD51 afectando a la funcionalidad de la proteína BRCA2 [44]. Por tanto, se debería considerar su posible patogenicidad de acuerdo a los estudios funcionales descritos en la bibliografía.
– La variante c.442A>G produce un cambio de arginina por glicina en el residuo 148 de la proteína CHEK2. Las herramientas *in silico* no predicen un impacto significativo en el *splicing*, a pesar de producirse el cambio a tres nucleótidos del final del segundo exón codificante. Sin embargo, la predicción computacional y un estudio funcional *in vivo* realizado en levaduras por Delimitsou A. y colaboradores sugieren que puede tener un impacto nocivo en la función de la proteína [45]. Además, debería considerarse su posible patogenicidad ya que en la bibliografía consultada se ha descrito en pacientes con antecedentes personales y/o familiares de cáncer de mama y de ovario hereditario [46].
– La variante c.1427C>T produce un cambio de treonina por metionina en el residuo 476 de la proteína CHEK2 y ha sido descrita en pacientes con cáncer de mama, pero también en cáncer colorrectal [47, 48]. La mayoría de las herramientas bioinformáticas y un estudio funcional *in vivo* realizado en levaduras por Roeb W. y colaboradores la predicen deletérea [49, 50]. Además, el fenotipo alarmante del caso índice perteneciente a nuestro estudio (cáncer de mama a los 21 años), justifica la necesidad confirmar su patogenicidad mediante un estudio de cosegregación de la variante.

## Supplementary Material

Supplementary Material

## Material Suplementario

Este artículo ofrece material suplementario (https://doi.org/10.1515/almed-2023-0032).

## References

[1] Sung H, Ferlay J, Siegel RL, Laversanne M, Soerjomataram I, Jemal A (2021). Global Cancer Statistics 2020: GLOBOCAN Estimates of Incidence and Mortality Worldwide for 36 Cancers in 185 Countries. CA Cancer J Clin.

[2] Ferlay J, Ervik M, Lam F, Colombet M, Mery L, Piñeros M (2020). Global Cancer Observatory: Cancer Today.

[3] Llort G, Chirivella I, Morales R, Serrano R, Sanchez AB, Teulé A (2015). SEOM clinical guidelines in Hereditary Breast and ovarian cancer. Clin Transl Oncol.

[4] Toss A, Tomasello C, Razzaboni E, Contu G, Grandi G, Cagnacci A (2015). Hereditary ovarian cancer: Not only BRCA1 and 2 genes. Biomed Res Int.

[5] Economopoulou P, Dimitriadis G, Psyrri A (2015). Beyond BRCA: new hereditary breast cancer susceptibility genes. Cancer Treat Rev.

[6] Eccles DM, Mitchell G, Monteiro ANA, Schmutzler R, Couch FJ, Spurdle AB (2015). BRCA1 and BRCA2 genetic testing-pitfalls and recommendations for managing variants of uncertain clinical significance. Ann Oncol.

[7] Bonache S, Esteban I, Moles-Fernández A, Tenés A, Duran-Lozano L, Montalban G (2018). Multigene panel testing beyond BRCA1/2 in breast/ovarian cancer Spanish families and clinical actionability of findings. J Cancer Res Clin Oncol.

[8] Rebbeck TR, Friebel TM, Friedman E, Hamann U, Huo D, Kwong A (2018). Mutational spectrum in a Worldwide study of 29,700 families with BRCA1 or BRCA2 mutations. Hum Mutat.

[9] Vega A, Campos B, Bressac-de-Paillerets B, Bond PM, Janin N, Douglas FS (2001). The R71G BRCA1 is a founder Spanish mutation and leads to aberrant splicing of the transcript. Hum Mutat.

[10] Díez O, Osorio A, Robledo M, Barroso A, Domènech M, Cortés J (1999). Prevalence of BRCA1 and BRCA2 Jewish mutations in Spanish breast cancer patients. Br J Cancer.

[11] Sagi M, Eilat A, Ben Avi L, Goldberg Y, Bercovich D, Hamburger T (2011). Two BRCA1/2 founder mutations in Jews of Sephardic origin. Fam Cancer.

[12] Tuazon AMA, Lott P, Bohórquez M, Benavides J, Ramirez C, Criollo A (2020). Haplotype analysis of the internationally distributed BRCA1 c.3331_3334delCAAG founder mutation reveals a common ancestral origin in Iberia. Breast Cancer Res.

[13] Neuhausen SL, Godwin AK, Gershoni-Baruch R, Schubert E, Garber J, Stoppa-Lyonnet D (1998). Haplotype and phenotype analysis of nine recurrent BRCA2 mutations in 111 families: results of an international study. Am J Hum Genet.

[14] Diez O, Gutiérrez-Enríquez S, Balmaña J (2010). Heterogeneous prevalence of recurrent BRCA1 and BRCA2 mutations in Spain according to the geographical area: implications for genetic testing. Fam Cancer.

[15] González-Santiago S, Ramón y Cajal T, Aguirre E, Alés-Martínez JE, Andrés R, Balmaña J (2020). SEOM clinical guidelines in hereditary breast and ovarian cancer (2019). Clin Transl Oncol.

[16] Daly MB, Pilarski R, Berry MP, Buys SS, Friedman S, Garber JE (2019). NCCN clinical practice guidelines in oncology. Genetic/familial high-risk assessment: breast, ovarian, and pancreatic. J Natl Compr Canc Netw.

[17] Richards S, Aziz N, Bale S, Bick D, Das S, Gastier-Foster J (2015). Standards and guidelines for the interpretation of sequence variants: a joint consensus recommendation of the American College of medical Genetics and Genomics and the association for molecular pathology. Genet Med.

[18] Machado PM, Brandão RD, Cavaco BM, Eugénio J, Bento S, Nave M (2007). Screening for a BRCA2 rearrangement in high-risk breast/ovarian cancer families: evidence for a founder effect and analysis of the associated phenotypes. J Clin Oncol.

[19] Cunningham F, Allen JE, Allen J, Alvarez-Jarreta J, Amode MR, Armean IM (2022). Ensembl 2022. Nucleic Acids Res.

[20] Vreeswijk MPG, Kraan JN, van der Klift HM, Vink GR, Cornelisse CJ, Wijnen JT (2009). Intronic variants in BRCA1 and BRCA2 that affect RNA splicing can be reliably selected by splice-site prediction programs. Hum Mutat.

[21] Ioannidis NM, Rothstein JH, Pejaver V, Middha S, McDonnell SK, Baheti S (2016). REVEL: an Ensemble method for predicting the pathogenicity of Rare missense variants. Am J Hum Genet.

[22] PubMed [Internet] (1996). Bethesda (MD): National Center for Biotechnology Information (U.S. National Institutes of Health’s National Library of Medicine).

[23] Kopanos C, Tsiolkas V, Kouris A, Chapple CE, Albarca Aguilera M, Meyer R (2019). VarSome: the human genomic variant search engine. Bioinformatics.

[24] Buys SS, Sandbach JF, Gammon A, Patel G, Kidd J, Brown KL (2017). A study of over 35,000 women with breast cancer tested with a 25-gene panel of hereditary cancer genes. Cancer.

[25] Rosado-Jiménez L, Mestre-Terkemani Y, Garcia-Hernández R, Zafra-Poves M, García-Aliaga A, Expósito-Garcia M (2021). Prevalence and phenotype, of the most frequent BRCA1/BRCA2 mutations, related to the hereditary breast and ovarian cancer syndrome, in families from Murcia (south-east of Spain). Clin Chem Lab Med.

[26] Hoang LN, Gilks BC (2018). Hereditary breast and ovarian cancer syndrome: moving beyond BRCA1 and BRCA2. Adv Anat Pathol.

[27] Swain SM, Wilson JW, Mamounas EP, Bryant J, Wickerham DL, Fisher B (2004). Estrogen receptor status of primary breast cancer is predictive of estrogen receptor status of contralateral breast cancer. J Natl Cancer Inst.

[28] Gutiérrez-Enríquez S, de La Hoya M, Martínez-Bouzas C, de Abajo AS, Cajal TRy., Llort G (2007). Screening for large rearrangements of the BRCA2 gene in Spanish families with breast/ovarian cancer. Breast Cancer Res Treat.

[29] de la Hoya M, Gutiérrez-Enríquez S, Velasco E, Osorio A, de Abajo AS, Vega A (2006). Genomic rearrangements at the BRCA1 locus in Spanish families with breast/ovarian cancer. Clin Chem.

[30] Gabaldó-Barrios X, Sarabia-Meseguer MD, Alonso-Romero JL, Marín-Vera M, Marín-Zafra G, Sánchez-Henarejos P (2014). Novel BRCA1 deleterious mutation (c.1918C>T) in familial breast and ovarian cancer syndrome who share a common ancestry. Fam Cancer.

[31] Castillo-Guardiola V (2019). Aportación de la secuenciación masiva de nueva generación en el diagnóstico del Síndrome de Cáncer de Mama y Ovario Hereditario mediante el uso de un panel de genes.

[32] Rosado-Jiménez L, Mestre-Terkemani Y, García-Aliaga A, Garcia-Hernández R, Zafra-Poves M, Expósito-Garcia M (2021). Prevalence and founder effect of the BRCA1 c.1918C>T variant in hereditary breast and ovarian cancer families from Murcia (southeastern Spain). Clin Chem Lab Med.

[33] Ruiz de Garibay G, Gutiérrez-Enríquez S, Garre P, Bonache S, Romero A, Palomo L (2012). Characterization of four novel BRCA2 large genomic rearrangements in Spanish breast/ovarian cancer families: review of the literature, and reevaluation of the genetic mechanisms involved in their origin. Breast Cancer Res Treat.

[34] Rosado-Jiménez L, Mestre-Terkemani Y, García-Aliaga A, Sánchez-Henarejos P, Macías-Cerrolaza JA, Moya-Martínez P (2021). Evidence for a founder effect of BRCA2 rearrangement in Spanish hereditary breast/ovarian cancer families. Clin Chem Lab Med.

[35] Mahecha D, Nuñez H, Lattig MC, Duitama J (2022). Machine learning models for accurate prioritization of variants of uncertain significance. Hum Mutat.

[36] Mersch J, Brown N, Pirzadeh-Miller S, Mundt E, Cox HC, Brown K (2018). Prevalence of variant reclassification following hereditary cancer genetic testing. JAMA.

[37] Li A, Swift M (2000). Mutations at the ataxia-telangiectasia locus and clinical phenotypes of A-T patients. Am J Med Genet.

[38] George Priya Doss C, Rajith B (2012). Computational refinement of functional single nucleotide polymorphisms associated with ATM gene. PloS One.

[39] Carranza D, Vega AK, Torres-Rusillo S, Montero E, Martinez LJ, Santamaría M (2017). Molecular and functional characterization of a cohort of Spanish patients with ataxia-telangiectasia. Neuromolecular Med.

[40] Fiévet A, Bellanger D, Rieunier G, Dubois d’Enghien C, Sophie J, Calvas P (2019). Functional classification of ATM variants in ataxia-telangiectasia patients. Hum Mutat.

[41] Mangone FR, Miracca EC, Feilotter HE, Mulligan LM, Nagai MA (2015). ATM gene mutations in sporadic breast cancer patients from Brazil. SpringerPlus.

[42] Podralska M, Ziółkowska-Suchanek I, Żurawek M, Dzikiewicz-Krawczyk A, Słomski R, Nowak J (2018). Genetic variants in ATM, H2AFX and MRE11 genes and susceptibility to breast cancer in the polish population. BMC Cancer.

[43] Thorstenson YR, Roxas A, Kroiss R, Jenkins MA, Yu KM, Bachrich T (2003). Contributions of ATM mutations to familial breast and ovarian cancer. Cancer Res.

[44] Ochiai K, Yoshikawa Y, Yoshimatsu K, Oonuma T, Tomioka Y, Takeda E (2011). Valine 1532 of human BRC repeat 4 plays an important role in the interaction between BRCA2 and RAD51. FEBS Lett.

[45] Delimitsou A, Fostira F, Kalfakakou D, Apostolou P, Konstantopoulou I, Kroupis C (2019). Functional characterization of CHEK2 variants in a Saccharomyces cerevisiae system. Hum Mutat.

[46] Apostolou P, Dellatola V, Papadimitriou C, Kalfakakou D, Fountzilas E, Faliakou E (2021). CHEK2 pathogenic variants in Greek breast cancer patients : evidence for strong associations with estrogen receptor positivity, overuse of risk-reducing procedures and population founder effects. Cancers.

[47] Yurgelun MB, Kulke MH, Fuchs CS, Allen BA, Uno H, Hornick JL (2017). Cancer susceptibility gene mutations in individuals with colorectal cancer. J Clin Oncol.

[48] Eliade M, Skrzypski J, Baurand A, Jacquot C, Bertolone G, Loustalot C (2016). The transfer of multigene panel testing for hereditary breast and ovarian cancer to healthcare: what are the implications for the management of patients and families?. Oncotarget.

[49] Desrichard A, Bidet Y, Uhrhammer N, Bignon YJ (2011). CHEK2 contribution to hereditary breast cancer in non-BRCA families. Breast Cancer Res.

[50] Roeb W, Higgins J, King MC (2012). Response to DNA damage of CHEK2 missense mutations in familial breast cancer. Hum Mol Genet.

