# Supplementary material for: Variantes genéticas recurrentes y priorización de variantes de significado clínico desconocido asociadas al síndrome de cáncer de mama y ovario hereditario en familias de la Región de Murcia
Source: Adv Lab Med. 2023 Jul 10;4(3):288–97. [Article in Spanish] doi: 10.1515/almed-2023-0032 (PMC10701495; doi:10.1515/almed-2023-0032)

**Tabla suplementaria 1.** Variantes patogénicas *BRCA1/2* agrupadas por región geográfica

**
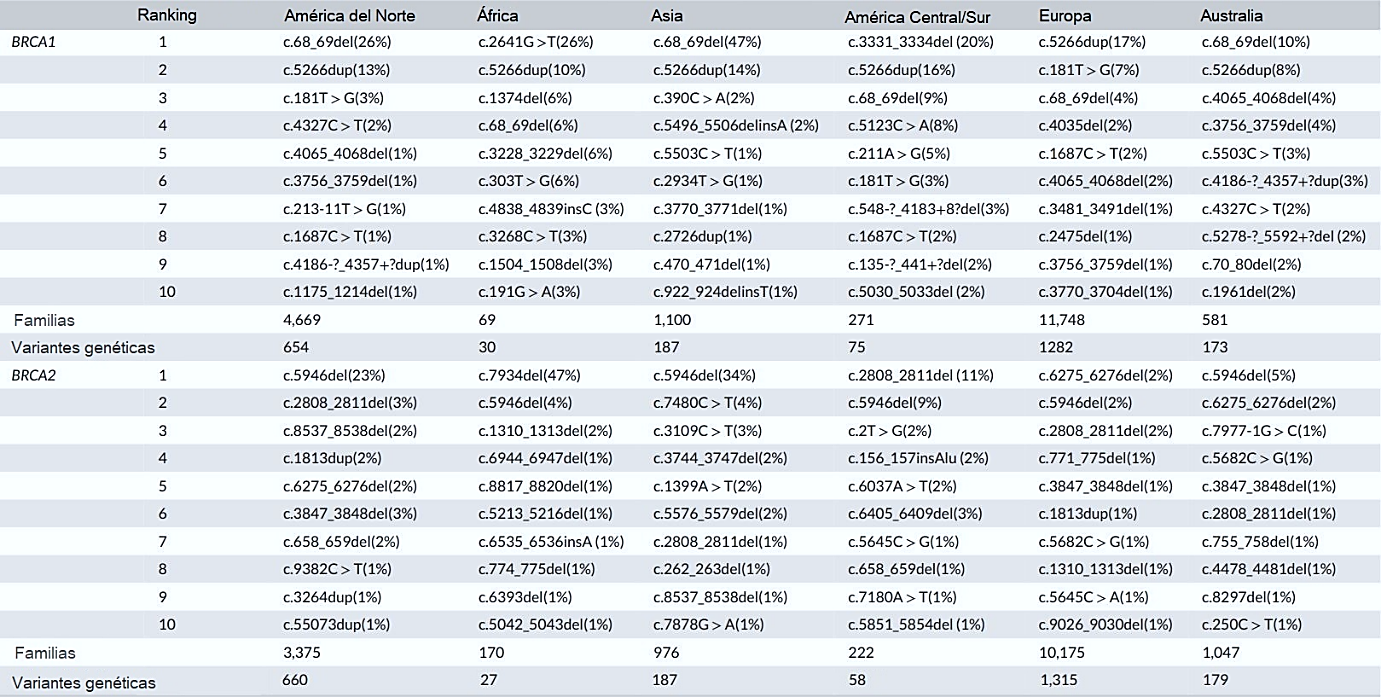
**

**Tabla suplementaria 2.** Variantes patogénicas *BRCA1/2* agrupadas por grupo racial/etnia

**
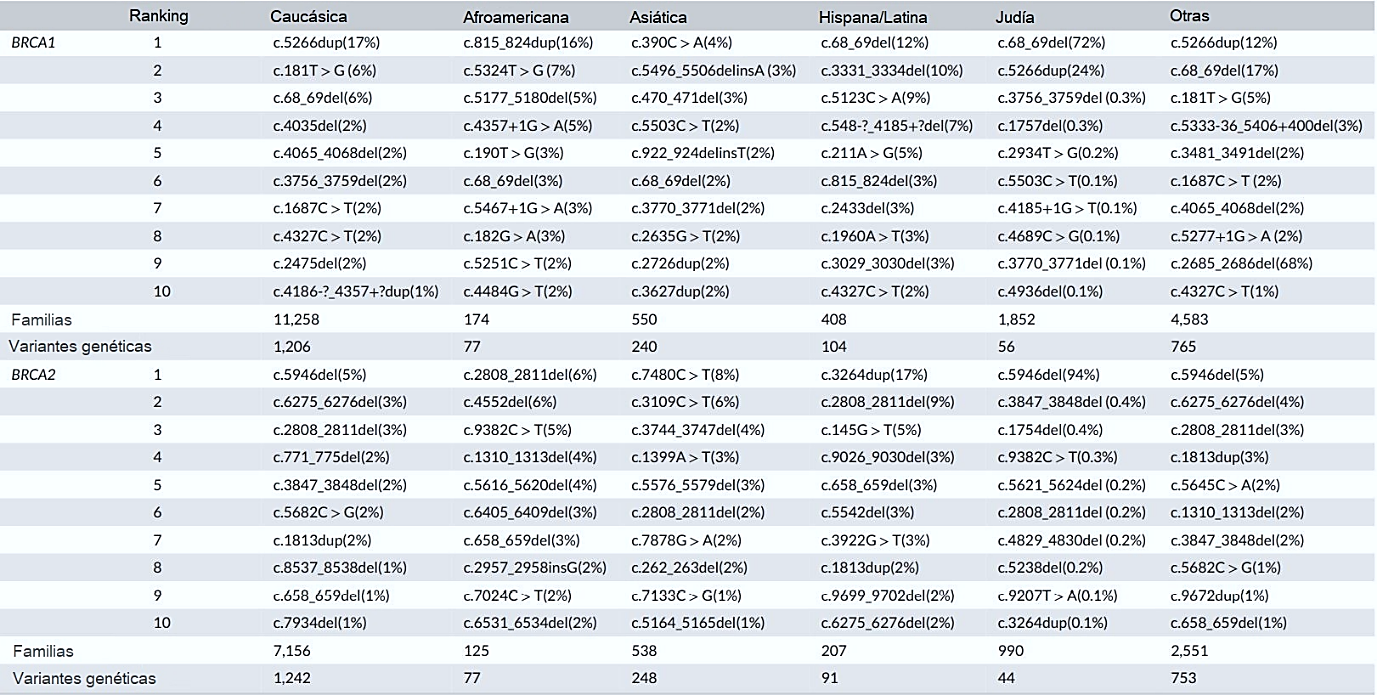
**

**Tabla suplementaria 3.** Evolución de criterios aplicados en el estudio genético del SCMOH

1. Criterios establecidos por la SEOM para estudio genético de *BRCA1* y *BRCA2* [3]

| Criterios independientes de la historia familiar |
| --- |
| Mujeres con cáncer de mama y cáncer de ovario sincrónico o metacrónico |
| Diagnóstico de CM a los 35 años o antes (40 años si se trata de una familia con menos de dos mujeres que sigan vivas a la edad de 45 años en cada rama de la familia) |
| CM bilateral (el primero diagnosticado a los 40 años o antes) |
| CM triple negativo diagnosticado a los 50 años o antes. |
| Diagnóstico de CO de alto grado epitelial no mucinoso (o de las trompas de Falopio o cáncer peritoneal primario). |
| 2 o más familiares de primer grado con cualquiera de las siguientes combinaciones |
| Cáncer de mama bilateral y otro cáncer antes de los 50 años |
| Cáncer de mama en el varón |
| Cáncer de mama y cáncer de ovario |
| Dos casos de cáncer de mama diagnosticados antes de los 50 años |
| 3 o más familiares directos de la misma rama familiar con cáncer de mama y / o cáncer de ovario |

1. Criterios de inclusión consensuados por el Comité de Asesoramiento Genético de la Región de Murcia para el estudio genético del SCMOH mediante panel de genes

| Análisis genético para *BRCA1* y *BRCA2* no informativo más alguno de los siguientes criterios que tienen en cuenta la historia familiar de cáncer |
| --- |
| Tres o más individuos de dos generaciones distintas que están afectados por cáncer de mama u ovario. Al menos dos individuos deben haber sido diagnosticados antes de los 50 años o uno antes de los 40. |
| Dos o más individuos han sido diagnosticados de cáncer de mama u ovario antes de los 35 años |
| Un caso de cáncer de mama en el varón y otro de mama u ovario antes de los 50 años. |
| Un caso de ovario antes de los 35 años y un caso de cáncer de mama antes de los 50 años en un familiar de primer grado. |
| Análisis genético para *BRCA1* y *BRCA2* no informativo más alguno de los siguientes criterios que tienen en cuenta la historia personal de cáncer |
| Diagnóstico de cáncer de mama y ovario en la misma paciente, siempre y cuando uno sea diagnosticado antes de los 50 años. |
| Un único individuo ha sido diagnosticado de cáncer de mama u ovario antes de los 25 años. |

1. Criterios actuales establecidos por la SEOM para la selección de pacientes de alto riesgo candidatos al estudio genético del SCMOH [15]

| Criterios independientes de la historia familiar |
| --- |
| Mujeres con cáncer de mama y cáncer de ovario sincrónico o metacrónico. |
| Cáncer de mama ≤ 40 años |
| Cáncer de mama bilateral (el primero diagnosticado ≤ 40 años) |
| Cáncer de mama triple negativo diagnosticado ≤ 60 años. |
| Diagnóstico de cáncer de ovario de alto grado epitelial no mucinoso (o de las trompas de Falopio o cáncer peritoneal primario). |
| Ascendencia con mutaciones fundadoras |
| Mutación somática BRCA detectada en cualquier tipo de tumor con una frecuencia alélica > 30% (si se conoce) |
| Pacientes con cáncer de mama metastásico HER2 negativo elegibles para considerar la terapia con inhibidores de PARP |
| 2 o más familiares de primer grado con cualquier combinación de las siguientes características de alto riesgo |
| Cáncer de mama bilateral y otro cáncer antes de los 60 años |
| Cáncer de mama <50 años y cáncer de próstata o páncreas <60 años |
| Cáncer de mama en el varón |
| Cáncer de mama y cáncer de ovario |
| Dos casos de cáncer de mama diagnosticados antes de los 50 años. |
| 3 o más familiares directos con cáncer de mama (al menos una premenopáusica) y / o cáncer de ovario y / o cáncer de páncreas o cáncer de próstata de Gleason alto (≥ 7) |

**Tabla suplementaria 4.** Reacción de amplificación.** kit GoTaq® Hot Start Polymerase-PCR-Promega;* F: *forward*, *R: reverse.*

| PCR amplificación | Volumen (µL) |
| --- | --- |
| H_2_O | 2 |
| *Master mix** | 6,25 |
| Cebador F +R (2 µM) | 1,25 |
| *Q-solution** | 2,5 |
| ADN (25 ng/µL) | 0,5 |
| Volumen final | 12,5 µL |

**Figura suplementaria 1.** Programa empleado en el termociclador *Veriti HID 96-Well ThermalCycler (Applied Biosystems)* para la PCR de amplificación.

Tras la amplificación, se llevó a cabo la electroforesis capilar en el analizador ABI3130 mediante análisis de fragmentos. Para ello, se mezcló 1μl del producto de amplificación con 12 μl de formamida Hi-Di y 0.5 μl del marcador de tamaño LIZ-500 (*Gene Scan-500 LIZ Size Standard-Applied Biosystems- Fisher Scientific)* y se desnaturalizó durante 5 minutos a 95º.

Los datos obtenidos tras la electroforesis capilar se analizaron en el *software* *Gene Mapper* v 4.0 facilitado por *Applied Biosystems*


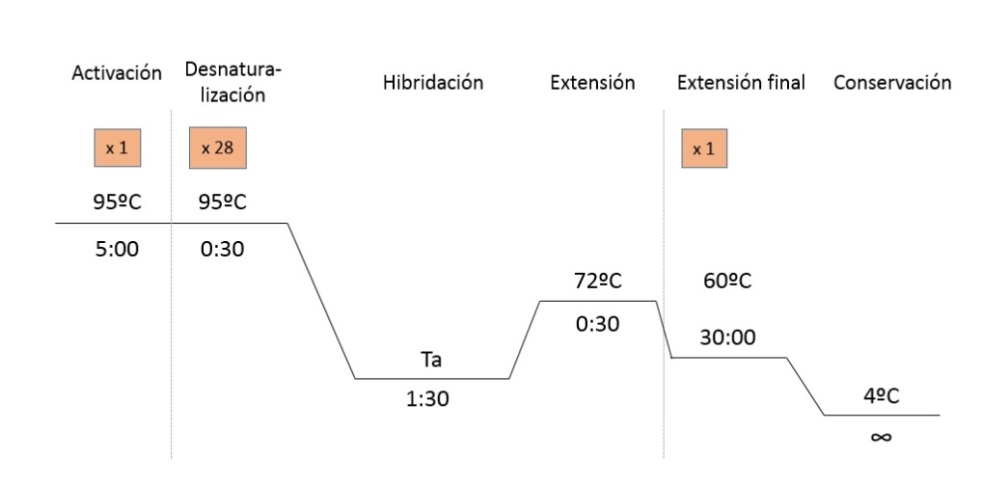


**Tabla suplementaria 5.** Secuencias de los cebadores empleados en el análisis de haplotipo de la variante c.1918C>T en *BRCA1* (Los cebadores *forward* se marcaron con el fluoróforo FAM para su empleo en el análisis de fragmentos). Ta: temperatura *annealing* o de anillamiento, pb: pares de bases

| MARCADORES MICROSATÉLITES EN *BRCA1* | | | | | |
| --- | --- | --- | --- | --- | --- |
| Marcador | | Cebador *forward* | Cebador *reverse* | Ta (°C) | Tamaño (pb) |
| M1 | D17S855 | [6FAM] GGATGGCCTTTTAGAAAGTGG | ACACAGACTTGTCCTACTGCC | 58 | 139-152 |
| M2 | 17-3858 | [6FAM] CATTTGCTGTTCCCTACCCCACAC | AGAACAATGCAAATTGAAGCAATGA | 58 | 127-134 |
| M3 | D17S1326 | [6FAM] CAGCTGATATTTCACAGGACT | AGAGCAAAACTCCATCTCAAACA | 58 | 89-108 |
| M4 | 17-3930 | [6FAM] ATCTTTGCCTAATCCAGGGTCACAAG | CAAATGGTGCTGGAATAGTTGGA | 57 | 286-298 |

**Figura suplementaria 2**. Localización de los marcadores microsatélites empleados con respecto al gen *BRCA1*


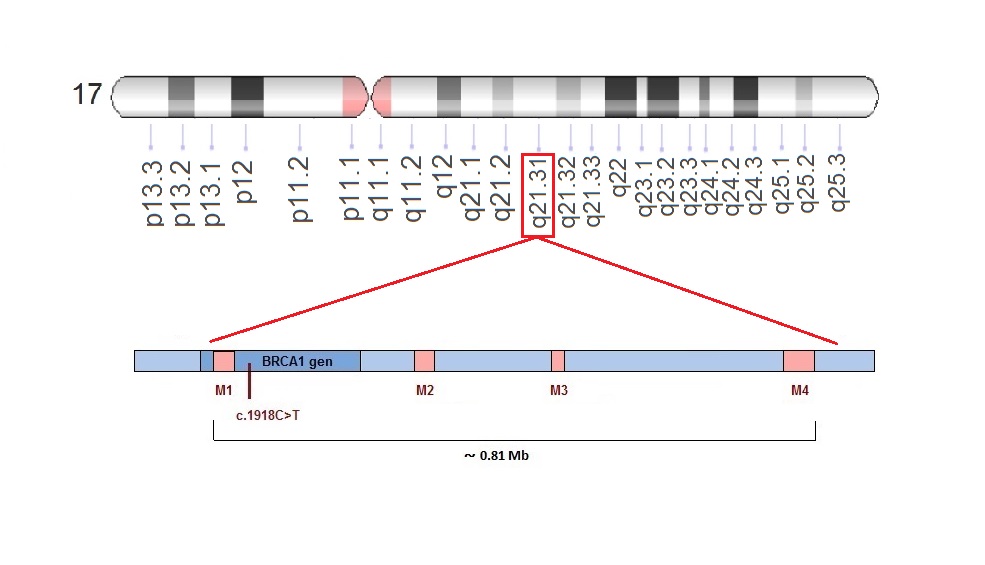


**Tabla suplementaria 6.** Secuencias de los cebadores empleados en el análisis de haplotipo de la deleción del exón 2 en *BRCA2* (Los cebadores *forward* se marcaron con el fluoróforo FAM para su empleo en el análisis de fragmentos) Ta: temperatura *annealing* o de anillamiento, pb: pares de bases

| MARCADORES MICROSATÉLITES EN *BRCA2* | | | | | |
| --- | --- | --- | --- | --- | --- |
| Marcador | | Cebador *forward* | Cebador *reverse* | Ta (°C) | Tamaño (pb) |
| M1 | D13S260 | [6FAM] AGATATTGTCTCCGTTCCATGA | CCCAGATATAAGGACCTGGCTA | 57 | 158 - 173 |
| M2 | D13S1493 | [6FAM] ACCTGTTGTATGGCAGCAGT | GGTTGACTCTTTCCCCAACT | 57 | 223 - 248 |
| M3 | D13S153 | [6FAM] AGCATTGTTTCATGTTGGTG | CAGCAGTGAAGGTCTAAGCC | 58 | 212 - 236 |

**Figura suplementaria 3.** Localización de los marcadores microsatélites empleados con respecto al gen *BRCA2*


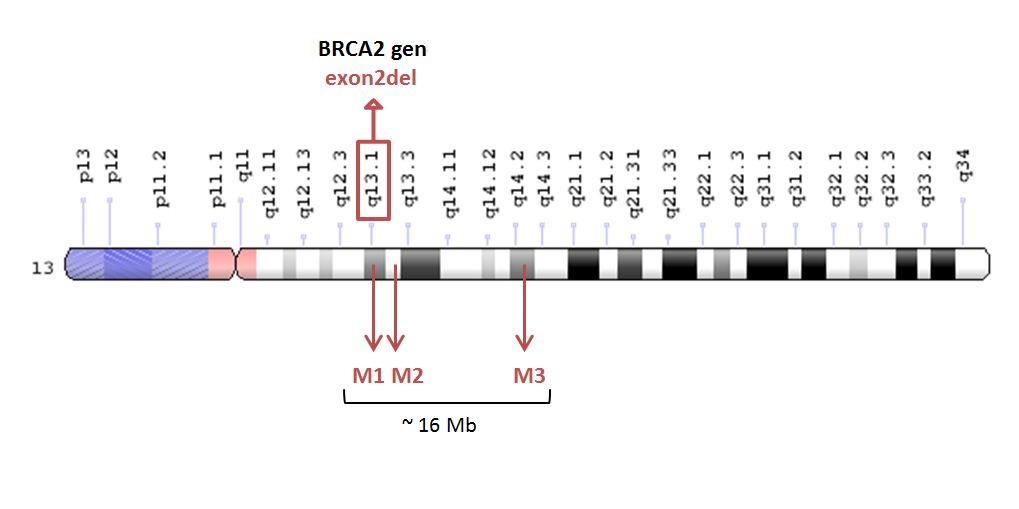


**Tabla suplementaria 7.** Secuencias de los cebadores empleados en el análisis de haplotipo de la variante c.8251_8254del en *ATM* (Los cebadores *forward* se marcaron con el fluoróforo FAM para su empleo en el análisis de fragmentos) Ta: temperatura *annealing* o de anillamiento, pb: pares de bases

| MARCADORES MICROSATÉLITES EN *ATM* | | | | | |
| --- | --- | --- | --- | --- | --- |
| Marcador | | Cebador *forward* | Cebador *reverse* | Ta (°C) | Tamaño (pb) |
| M1 | D11S4078 | [6FAM] AGGCTAACCAGCCAACATT | CGGGCTATAAAACTCAAGTCTC | 61 | 135-159 |
| M2 | D11S1391 | [6FAM] TGCATGCATACATACATACATACA | CATCCATCCCTCTGTCTCTG | 55 | 158-178 |
| M3 | D11S1781 | [6FAM] AGCTGTTCTTGTCACAGGAGAG | ACAAATTGTCAGTGCCCC | 55 | 243-251 |
| M4 | D11S1390 | [6FAM] GGGTGGAATCCTTCAGAATT | AAATATTACCGGGCTTGGAC | 57 | 145-165 |
| M5 | D11S4176 | [6FAM] AGACTCTCTCGTCCTCAGGG | GGGTAGCACTCCCAGGTT | 62 | 224-254 |
| M6 | D11S4197 | [6FAM] TGAGGTCAATGTTGGTTTC | TAGTAGAATCTCATAGGTTCTGTGG | 57 | 227-271 |

**Figura suplementaria 4.** Localización de los marcadores microsatélites empleados con respecto al gen *ATM*


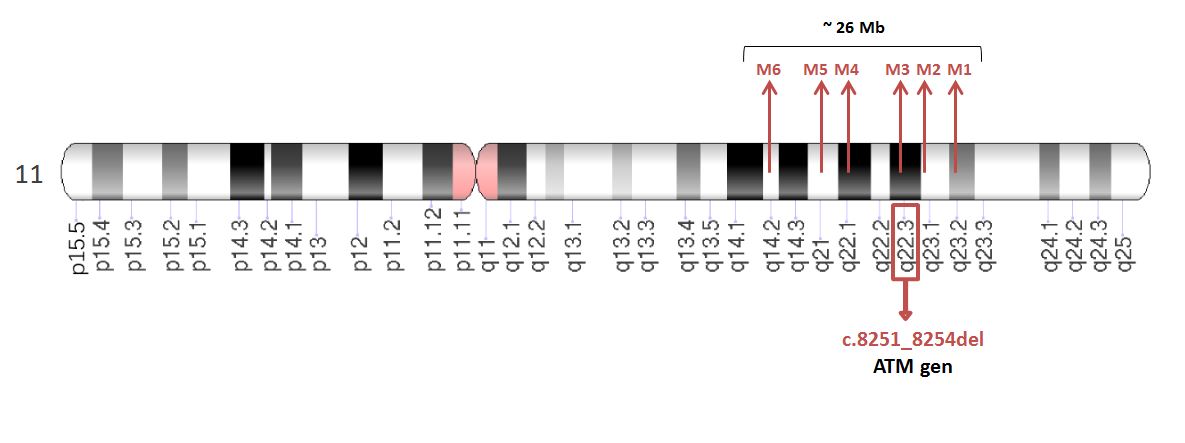


**Tabla suplementaria 8.** Haplotipos obtenidos en los portadores de la variante c.1918C>T en *BRCA1.* (El haplotipo común aparece resaltado en verde) CI: caso índice, M: microsatélite

|  | M1 (D17S855) | | M2 (17-3858) | | M3 (D17S1326) | | M4 (17-3930) | |
| --- | --- | --- | --- | --- | --- | --- | --- | --- |
| CI 1 | 139 | 144 | 122 | 126 | 89 | 108 | 308 | 298 |
| CI 2 | 139 | 146 | 122 | 126 | 89 | 106 | 308 | 298 |
| CI 3 | 139 | 146 | 122 | 114 | 89 | 102 | 308 | 301 |
| CI 4 | 139 | 144 | 122 | 128 | 89 | 106 | 308 | 298 |
| CI 5 | 139 | 148 | 122 | 134 | 89 | 106 | 308 | 286 |
| CI 6 | 139 | 146 | 122 | 128 | 89 | 106 | 301 | 298 |
| CI 7 | 139 | 146 | 122 | 118 | 89 | 89 | 308 | 289 |
| CI 8 | 139 | 144 | 122 | 120 | 89 | 89 | 308 | 295 |

**Tabla suplementaria 9.** Haplotipos obtenidos en los portadores de la deleción del exón 2 en *BRCA2.* (El haplotipo común aparece resaltado en verde) CI: caso índice, M: microsatélite

|  | M1 (D13S260) | | M2 (D13S1493) | | M3 (D13S153) | |
| --- | --- | --- | --- | --- | --- | --- |
| CI 1 | 161 | 167 | 223 | 227 | 223 | 219 |
| CI 2 | 161 | 160 | 223 | 235 | 226 | 226 |
| CI 3 | 161 | 167 | 227 | 231 | 221 | 228 |
| CI 4 | 161 | 161 | 223 | 227 | 223 | 219 |
| CI 5 | 161 | 165 | 223 | 223 | 223 | 230 |
| CI 6 | 161 | 163 | 223 | 223 | 211 | 219 |
| CI 7 | 161 | 163 | 223 | 227 | 211 | 217 |
| CI 8 | 161 | 163 | 223 | 227 | 223 | 219 |
| CI 9 | 161 | 163 | 223 | 235 | 223 | 211 |
| CI 10 | 161 | 157 | 223 | 223 | 211 | 219 |
| CI 11 | 161 | 163 | 227 | 227 | 215 | 228 |
| CI 12 | 161 | 157 | 223 | 235 | 215 | 219 |

**Figura suplementaria 5.** Haplotipo de los portadores de la variante c.8251_8254del en *ATM.* (El haplotipo común aparece resaltado en verde) CM: cáncer de mama, CMb: cáncer de mama bilateral, CP: cáncer de páncreas, CPr: cáncer de próstata, CT: cáncer de tiroides, LCM: linfoma células del manto, Mel: melanoma


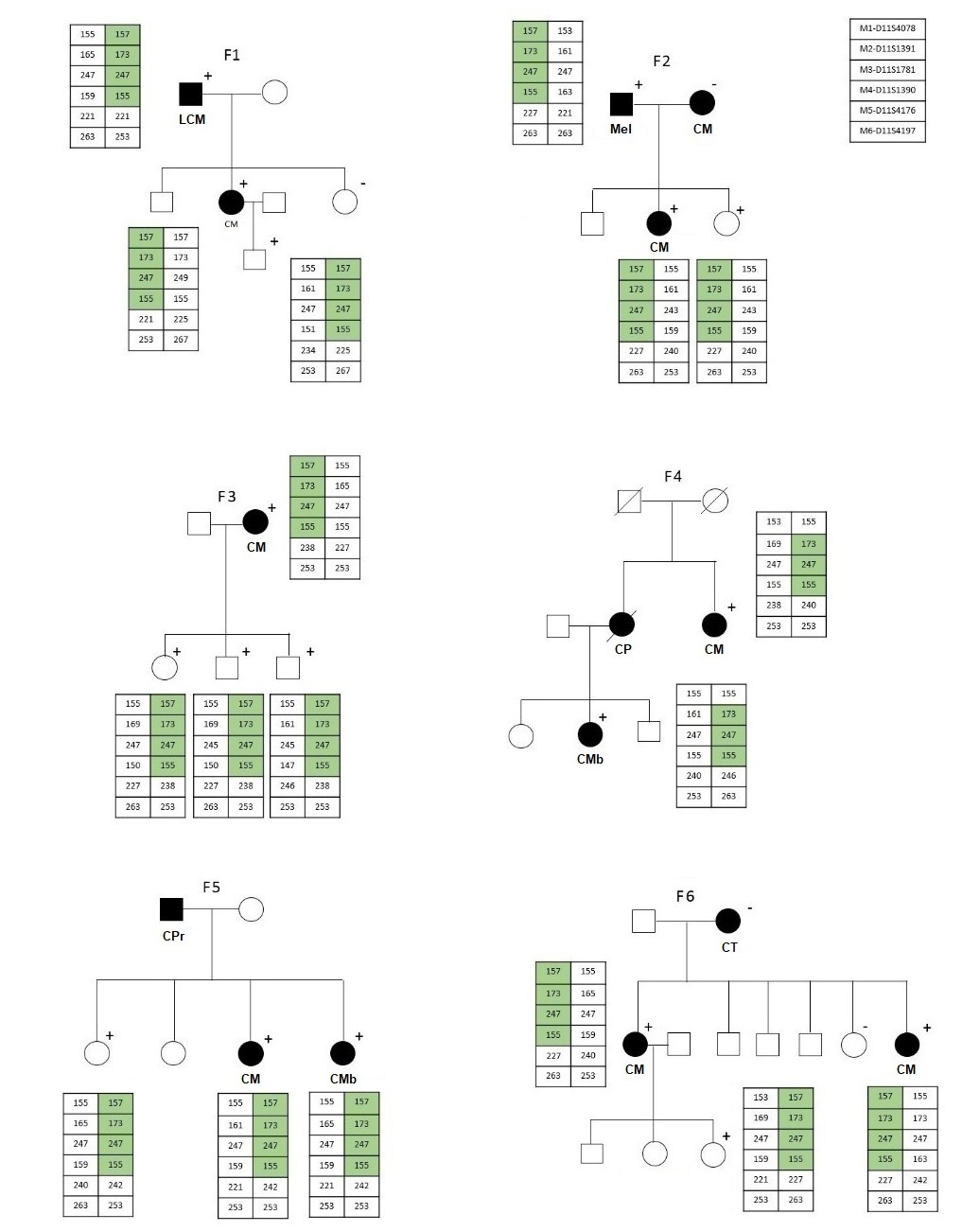

Supplement: Supplementary file 1 — Supplementary Material [file j_almed-2023-0032_suppl_001.docx]
